# Supplementary material for: Sleep Assessment in Patients with Inner Ear Functional Disorders: A Prospective Cohort Study Investigating Sleep Quality Through Polygraphy Recordings
Source: Audiol Res. 2025 Jun 24;15(4):76. doi: 10.3390/audiolres15040076 (PMC12286230; doi:10.3390/audiolres15040076)
Supplement: Supplementary file 1 [file audiolres-15-00076-s001.zip › audiolres-3656645-supplementary.pdf]

Supplementary Materials - Supplementary Table S1

Sleep parameter	Compared groups	*p-value*	Compared groups	*p-value*	Compared groups	*p-value*	Compared groups	*p-value*
TGF	A1 vs. C1	0.111	A1 vs. C2	0.306	A2 vs. C2	0.570	A2 vs. C1	0.289
AHI		0.069		0.100		0.405		0.249
cAHI		**0.049**		**0.041**		0.302		0.313
oAHI		0.119		0.275		0.790		0.483
AAI		0.603		0.778		0.473		0.667
AAIre		0.289		0.350		0.986		0.813
RERA		0.979		0.646		0.300		0.579
AAI-f		0.843		0.843		0.843		0.843
Sn		0.409		0.650		0.210		0.104
TGF	A1 vs. B1	0.384	A1 vs. B2	0.359	A2 vs. B2	0.606	A2 vs. B1	0.644
AHI		0.716		0.789		0.361		0.404
cAHI		0.843		0.338		0.602		0.405
oAHI		0.693		0.985		0.248		0.470
AAI		0.079		**0.022**		0.117		0.321
AAIre		0.127		0.185		0.496		0.262
RERA		0.391		0.194		0.384		0.729
AAI-f		0.692		0.065		0.065		0.692
Sn		0.800		0.878		0.377		0.740
TGF	B1 vs. C1	0.520	B1 vs. C2	0.938	B2 vs. C2	0.997	B2 vs. C1	0.596
AHI		0.129		0.184		0.166		0.117
cAHI		0.114		0.110		0.165		0.321
oAHI		0.261		0.390		0.210		0.136
AAI		0.089		**0.038**		**0.005**		**0.015**
AAIre		0.386		0.188		0.430		0.663
RERA		0.453		0.227		0.118		0.293
AAI-f		0.534		0.534		**0.028**		**0.028**
Sn		0.288		0.479		0.752		0.470

The p-value was based on a t-test for independent samples.

TGF; time with good flow signal quality, AHI; overall apnea-hypopnea index, cAHI; central apnea-hypopnea index, oAHI; obstructive apnea-hypopnea index, AAI; autonomic arousal index, AAIre; autonomic arousal index related to respiratory events, RERA; respiratory effort-related arousal index, AAI-f; risk of sleep fragmentation, Sn; snoring; A; acute idiopathic functional inner ear disease, B; chronic idiopathic functional inner ear disease, C; healthy controls without any otoneurological symptoms; 1; 1st night, 2; 2nd night
